# Melt Memory Effects in Poly(butylene succinate) Studied by Differential Fast Scanning Calorimetry

**DOI:** 10.3390/polym12122796

**Published:** 2020-11-26

**Authors:** Leire Sangroniz, Connie Ocando, Dario Cavallo, Alejandro J. Müller

**Affiliations:** 1 POLYMAT, Faculty of Chemistry, University of the Basque Country UPV/EHU, Paseo Manuel de Lardizábal, 3, 20018 Donostia-San Sebastián, Spain; connie.ocando@polymat.eu; 2 Department of Chemistry and Industrial Chemistry, University of Genova, via Dodecaneso 31, 16146 Genova, Italy; 3 IKERBASQUE, Basque Foundation for Science, 48013 Bilbao, Spain

**Keywords:** self-nucleation, melt memory, fast scanning calorimetry, poly(butylene succinate)

## Abstract

It is widely accepted that melt memory effect on polymer crystallization depends on thermal history of the material, however a systematic study of the different parameters involved in the process has been neglected, so far. In this work, poly(butylene succinate) has been selected to analyze the effect of short times and high cooling/heating rates that are relevant from an industrial point of view by taking advantage of fast scanning calorimetry (FSC). The FSC experiments reveal that the width of melt memory temperature range is reduced with the time spent at the self-nucleation temperature (*T_s_*), since annealing of crystals occurs at higher temperatures. The effectiveness of self-nuclei to crystallize the sample is addressed by increasing the cooling rate from *T_s_* temperature. The effect of previous standard state on melt memory is analyzed by (a) changing the cooling/heating rate and (b) applying successive self-nucleation and annealing (SSA) technique, observing a strong correlation between melting enthalpy or crystallinity degree and the extent of melt memory. The acquired knowledge can be extended to other semicrystalline polymers to control accurately the melt memory effect and therefore, the time needed to process the material and its final performance.

## 1. Introduction

The crystallization process of semicrystalline polymers depends on the thermal history of the material. To erase the thermal history, polymers have to be heated to 25–30 °C above their melting temperature. In this way, a homogeneous or isotropic melt is obtained. When the temperatures employed are high enough to produce an isotropic melt, during subsequent cooling in a differential scanning calorimeter (DSC), the material will always crystallize at the same temperature. This temperature is usually denoted as the standard crystallization temperature and it only depends on the cooling rate employed. Nevertheless, if the sample is heated to temperatures that are not high enough to reach an isotropic melt state (or erase melt memory), some self-seeds or self-nuclei survive, which can trigger crystallization at a higher temperature during the subsequent cooling run [1,2,3,4].

A thermal procedure known as self-nucleation (SN) was developed by Blundell et al. [1] and was modified to be employed in conventional DSC experiments by Fillon et al. [2]. When a material is completely molten, i.e., according to the DSC the sample is above the end of the melting endotherm, but the crystallization temperature is still higher than the standard crystallization temperature, it is said that the material has a melt memory effect. The nature of the melt memory effect has attracted much attention in the last few years, but it is still under debate [5,6,7,8,9,10,11,12]. In a recent review [4] some of us have analyzed the different theories and the parameters that affect melt memory, such as the molecular weight of the material [3,7,9,13], chain topology [13], the time spent at the self-nucleation temperature [3,7,14,15,16] and the effect of chemical structure [17] or confinement [18,19,20], among others. The new evidence about the nature of melt memory has been also summarized, which have been obtained taking advantage of new combined techniques for its study such as rheology [21,22,23], dielectric spectroscopy [24] or infrared spectroscopy [25,26]. 

Regarding the theories developed to explain melt memory effects, even though there is no consensus about it, some of them point towards a kinetic nature of melt memory, in the next lines some of these works are summarized. The works of Alamo and Hu [9,10] have shown that melt memory effects in random branched copolymers can result from the formation of a complex melt topology during the crystallization process, since sequences of appropriate crystallizable length have to diffuse from the melt to the crystal growth front. For some copolymers melt memory effects are observed even above the equilibrium melting temperature. Luo and Sommer [11] performed molecular dynamic studies concluding that melt memory could arise from disentangled regions of the melt. Lorenzo et al. [7] studied the rheological properties of PP at different self-nucleation temperatures stating that melt memory could arise from partial orientation of chain segments that were previously forming the crystal. Nevertheless, other authors consider that melt memory arises from the presence of small crystals that are not detected by DSC [2,8] or state that melt memory is an intermediate metastable melt state [12].

In any case, it is accepted that melt memory depends on the applied thermal history of the sample [3,4]. However, a thorough study analyzing systematically the different parameters that affect this phenomenon is missing, so far. In the literature, considerable work has been devoted to the effect of time spent at the self-nucleation temperature [3,4,5,6,7,14,15,16], with the aim of erasing the self-nuclei by spending long times at the *T_s_* temperature. Despite this, the effect of the time spent at a given *T_s_* temperature on *self-nucleation Domains*, and that of the cooling/heating rates employed, are not known. Considering that in industry short times and high cooling/heating rates are common to process polymers, it is of paramount importance to ascertain the effect of those parameters on self-nucleation.

Accessing high cooling/heating rates and short times in conventional DSC is not possible, as heating/cooling rates are below 100 K/min. The development of fast scanning chip calorimeter however, allows to study high cooling and heating rates, in the order of several thousands K/min, which are relevant from a processing and an academic point of view. It should be considered that the heating/cooling rate affects the thermal properties of the material and thus, its final performance. From an academic point of view, this technique enables to study the crystallization properties at high undercooling and to investigate the homogeneous nucleation of materials with fast crystallization kinetic, among other topics [27,28].

Although most of the research has been focused on the basic aspects of melt memory, recently practical applications of this phenomenon have been reported in literature. An interesting application of self-nucleation has been reported by Yuan et al. where they report improvements in the shape memory effect of chemically cross-linked polycaprolactone by taking advantage of a self-nucleation procedure [29]. Zhou et al. improved the mechanical properties of PP/LDPE binary blends reinforced with cold-drawn fibers, which act as self-seeds [30]. The mentioned examples reflect the importance of understanding the different parameters that affect the self-nucleation procedure or melt memory effect.

To determine the role of the different parameters involved in melt memory effects, in this work poly(butylene succinate) has been selected since it has a wide *melt memory Domain* [3,4,17,31], which enables to highlight small variations when the sample is submitted to different thermal procedures. Therefore, we systematically study by differential fast scanning calorimetry (FSC), for the first time, the effects of the following variables on the self-nucleation behavior of PBS: (a) the time employed during the self-nucleation protocol, (b) the cooling rate and (c) the effect of the generated standard state before self-nucleation. The knowledge obtained on self-nucleation in this study can provide a guide to fine-tune the properties of the material, like the crystallinity level, and to reduce the processing time by increasing the crystallization rate.

## 2. Materials and Methods

Polybutylene succinate was synthetized by melt polycondensation employing tetrabutoxy titane (TBT) as a catalyst (Across, Geel, Belgium). First, the esterification of succinic acid and 1,4-butanediol (Sigma Aldrich, Madrid, Spain) was performed followed by the polycondensation under vacuum. The reaction was performed under nitrogen, heating first the flask to 190 °C for 2 h and then to 200 °C for another 2 h under atmospheric pressure. Then, to proceed with polycondensation, the catalyst was added to the flask and stirred for 1 h and 30 min under vacuum, the flask was heated to 230 °C for 1 h and finally to 250 °C for 4 h.

In order to purify the polymer, first the obtained material was dissolved in chloroform (Chem Lab, Zedelgem, Belgium) and was washed with hydrochloric acid solution (0.1 M) (Sigma Aldrich, Madrid, Spain). Afterwards, the catalyst was removed washing the solution twice with demineralized water and precipitated in excess cold heptane (Labscan, Bangkok, Thailand). The precipitated polymer was filtered and dried at 40 °C under vacuum until a constant weight was obtained [31,32]. The obtained PBS has a number average molar mass of 25,000 g/mol. The sample was cut down with a microtome to obtain the appropriate thickness to be analyzed in the flash calorimeter.

The melt memory effect of PBS was studied with a flash DSC 2+ (Mettler Toledo, Barcelona, Spain) chip calorimeter or differential fast scanning calorimeter. The equipment is connected to a Huber TC-100 intracooler (Huber, Offenburg, Germany). The sensor was first conditioned and calibrated, then a small sample was placed on the sensor and two heating and cooling steps were conducted to ensure good contact between the polymer and the sensor. The measurements were carried out under nitrogen atmosphere, with a flow rate of 80 mL/min to avoid degradation. The STARe software was used to analyze the data (Mettler Toledo, Barcelona, Spain). 

**Figure 1:**
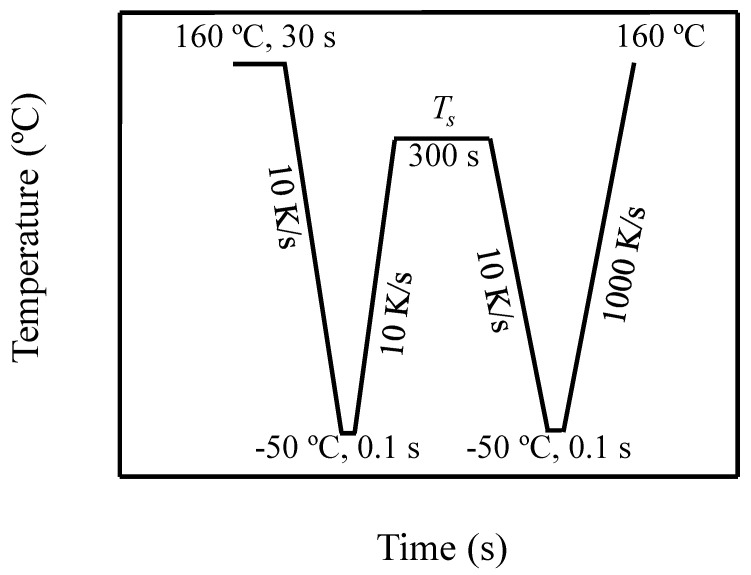
Standard self-nucleation procedure employed to investigate melt memory effects.

To study melt memory effects, the following self-nucleation procedure (see Figure 1), proposed by Fillon et al. [2,3], was employed: first the material is heated to 25–30 °C above its melting peak to erase the thermal history of the sample, in this case the sample was heated to 160 °C. Then the sample was cooled down to −50 °C to obtain a standard crystalline state and maintained at this temperature for 0.1 s. The sample was heated to the selected self-nucleation temperature, *T_s_*, and was maintained at this temperature for a certain period of time, usually 5 min. After that, the sample was cooled down and heated again. Depending on the conditions employed to perform the self-nucleation procedure and on the *T_s_* temperature, the sample can show three different *self-nucleation Domains*. The temperature region corresponding to each *Domain* can be determined by analyzing the cooling scan from the *T_s_* temperature and the subsequent heating scan. 

As a reference the results obtained applying a heating/cooling rate of 10 K/s were used, this rate was selected considering two factors: to measure the sample at the lowest rate possible without losing accuracy in the flash DSC, to allow the material to fully crystallize. The second factor considered was that no lower rates were employed since otherwise the sample spends longer times at high temperatures (as the measurement takes longer time), which might result in degradation.

In this work melting enthalpy values are discussed, which are directly correlated to the crystallinity level, since the mass of the sample employed in the flash DSC was not determined in this case.

## 3. Results

### 3.1. Self-Nucleation of PBS

**Figure 2:**
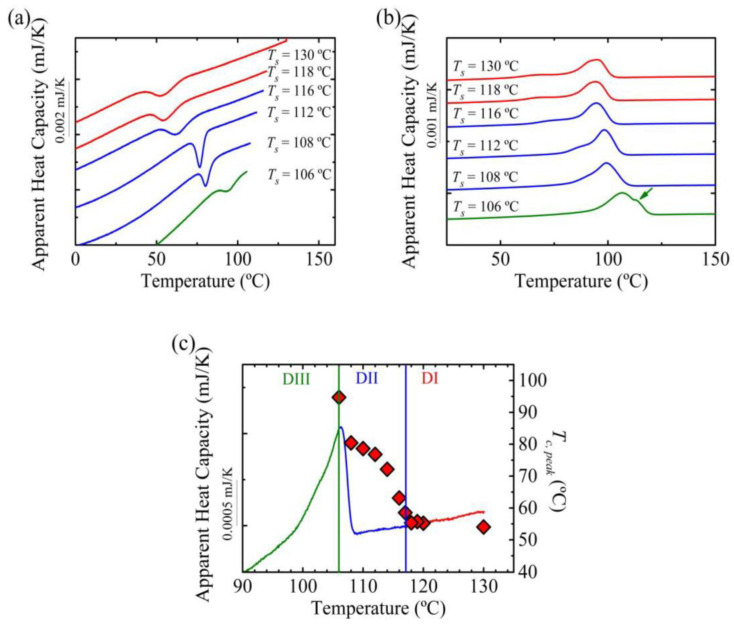
(**a**) Differential scanning calorimeter (DSC) cooling scans of PBS from the indicated self-nucleation temperatures and (**b**) subsequent heating scans. (**c**) Crystallization temperature (right-hand side *y*-axis) as a function of self-nucleation temperature (*x*-axis) on top of the PBS DSC heating scan (the DSC heating scan is represented in colors that match the different SN *Domains*).

Figure 2 shows the results obtained after applying a standard self-nucleation procedure (Figure 1). In this case, a cooling and heating rate of 10 K/s has been employed except in the final heating, in which 1000 K/s was used to avoid reorganization of crystals. The sample was kept at 0.1 s at the self-nucleation temperature. Under these conditions, the crystallization temperature was constant above *T_s_* temperatures equal to 118 °C. So, for temperatures equal or above 118 °C, the sample was in *Domain I* or the *melting Domain* [2,3,4]. However, when the *T_s_* temperature was reduced to 117 °C or below, an increase in the crystallization temperature with respect to the standard crystallization temperature was observed, which marks the transition to *Domain II* or the *self-nucleation Domain*. The enhancement of the crystallization temperature comes from the presence of self-nuclei and self-seeds, which increase the nucleation density.

PBS crystals were molten at 109 °C according to the FSC results (Figure 2), thus for temperatures above 109 °C there were no crystal fragments and consequently the increase of crystallization temperature in this region corresponded to the presence of self-nuclei; therefore, this temperature region is known as the *melt memory Domain* or *Domain IIa* [4,24], see Figure 2c. For temperatures below 109 °C, there are some crystal fragments (evidenced by incomplete melting in the DSC trace) that act as self-seeds responsible for the increase in crystallization temperature. This *T_s_* temperature range is called *Domain IIb* or the *self-seeding Domain* [4,24], see Figure 2c.

For self-nucleation temperatures equal or lower than 106 °C, if the subsequent heating scan is analyzed (Figure 2b), an additional melting peak is observed (signaled by an arrow), which corresponds to the melting of annealed crystals. The lower melting peak corresponds to less stable crystals with thin lamellae whereas the higher melting temperature corresponds to recrystallized or annealed crystals. During annealing the crystals reorganize and form more stable crystals, with thicker lamellae, which results in higher melting temperatures [33]. In Figure 2b a shift of the melting peak to higher temperatures is observed, in the case of the measurement in *Domain III* the lowest melting peak was considered (the highest melting peak corresponds to annealed crystals, as mentioned before). This shift resulted from the crystallization of the material at higher temperatures when cooling from *T_s_*, which led to crystals with thicker lamellae and thus, higher melting peaks. Summarizing, at temperatures equal or below 106 °C, the sample is in *Domain III* or the *self-nucleation and annealing Domain* [2,3,4].

Melt memory effects are considered to have a kinetic nature, since the temperature regions of the different *Domains* depend on the conditions employed to perform the experiment [2,3,4]. Although some aspects, such as the effect of time spent at *T_s_* temperature [3,7,14,15,16], have attracted attention in the literature, other aspects, such as the effect of cooling and heating rates have not been studied. In the following sections, the different parameters that affect the *self-nucleation Domains* are studied systematically to provide new insights into the control of melt memory effects by taking advantage of its kinetic nature.

### 3.2. Effect of Time Spent at the Self-Nucleation Temperature

Concerning the time effect on melt memory, different behaviors have been reported in the literature [3,5,6,7,14,15,16]. In a recent paper published by some of us [4], it is concluded that depending on the temperature range, different trends can be observed: for materials in *Domain III*, no effect of time has been observed. For samples in *Domain IIb* no effect of time or a slight reduction of crystallization temperature has been reported. Finally, for samples in *Domain IIa*, near *Domain I*, increasing the time spent at *T_s_* temperature, a reduction of crystallization temperature back to the standard crystallization temperature was reported. This means that when the sample is kept for long times at the right *T_s_* temperature (in *Domain IIa*, near *Domain I*), it is possible to erase all the self-nuclei, only if the temperature is close to *Domain I*. All these works have focused on the erasure of melt memory effects by increasing the time spent at *T_s_* temperatures and analyzing if the crystallization temperature is reduced due to the “dissolution” of self-nuclei or self-seeds. 

**Figure 3:**
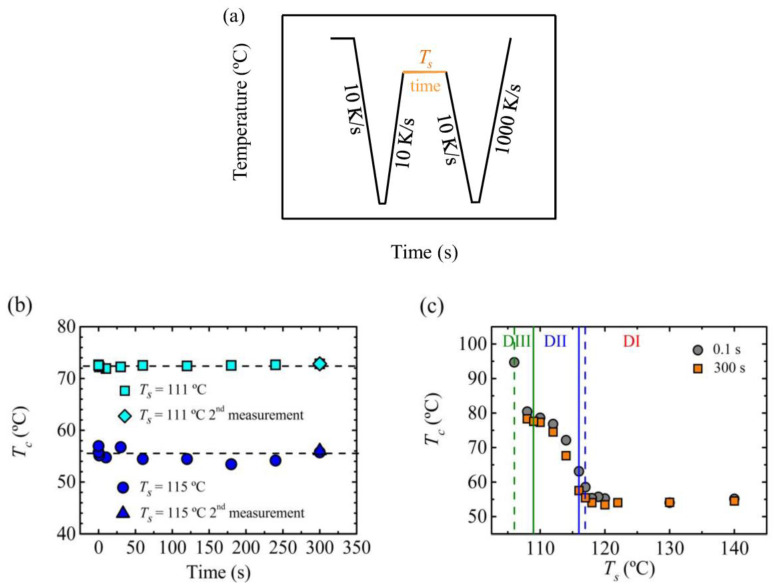
(**a**) Thermal procedure to analyze the effect of time spent at *T_s_* temperature, keeping the sample at about 160 °C for 30 s to erase completely the thermal history, and cooling down to −50 °C, the sample is kept at this temperature, −50 °C, for 0.1 s, (**b**) crystallization temperature corresponding to two *T_s_* temperatures of *Domain II* as a function of holding time at *T_s_* and (**c**) crystallization temperature as a function of *T_s_* temperature spending 0.1 s and 300 s at *T_s_* temperature, vertical lines indicate the transition temperature between *Domains*, dashed lines correspond to 0.1 s and solid lines to 300 s.

In this work, we studied the effect of time on crystallization temperature by varying the time from 0.1 to 300 s, for two selected *T_s_* temperatures that belong to *Domain II*: the lowest one corresponded to *DIIb*, and the highest one to *DIIa*. Figure 3b shows that for the lowest *T_s_*, the crystallization temperature is kept constant and independent of the time spent at *T_s_*. For the high *T_s_* temperature, there are some small variations, although a specific trend cannot be observed.

Figure 3c shows a plot of crystallization temperature versus *T_s_*, for two different holding times at the different *T_s_* values. The results superpose quite well, showing that there was no effect of time, in the range explored in this work, on the crystallization temperature.

It should be highlighted that in literature, only the effect of time on the subsequent crystallization temperature has been studied, but the effect of time on the transition temperature between *self-nucleation Domains* has not been reported. From a practical point of view, it is more interesting to study how keeping the sample for a very short time at a *T_s_* temperature, following the procedures involved in industrial processing, can alter the melt memory effect. 

**Figure 4:**
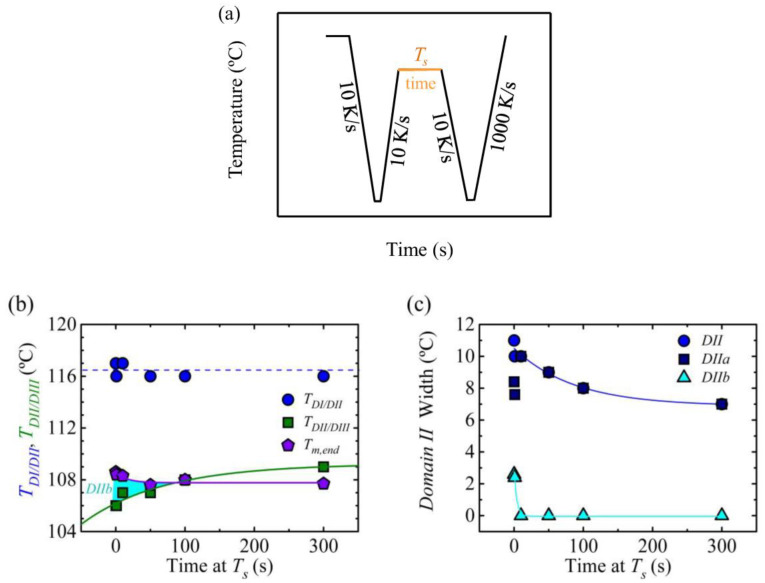
(**a**) Thermal procedure employed for varying the time spent at *T_s_*, keeping the sample at about 160 °C for 30 s to erase completely the thermal history, and cooling down to −50 °C, the sample is kept at this temperature, −50 °C, for 0.1 s, (**b**) transition temperature between *Domains* and temperature corresponding to the end of the melting endotherm (*T_m,end_*) and (**c**) width of *Domain II* as a function of time spent at *T_s_*.

Figure 4b shows the transition temperatures between different *Domains* when the sample was kept at the self-nucleation temperature between 0.1 and 300 s. The transition temperature between *Domain I* and *Domain II*, i.e., temperature of the self-nuclei’s ultimate stability, did not change with the time spent at *T_s_* temperature for the range of times analyzed in this work. On the other hand, the transition temperature between *Domain II* and *Domain III* increased with the time spent at *T_s_*. This means that when the sample is kept at *T_s_* for short times, lower temperatures are required to produce annealing of the crystal, because the short times spent at this temperature are not enough to anneal the small crystals that are left unmolten at this temperature. 

In Figure 4b, the temperature corresponding to the end of the melting endotherm was also displayed in the plot. It can be observed that this *T_m,end_* was maintained constant or at least the variation was within 1 °C. For times below 100 s, the transition temperature between *Domain II* and *Domain III* was lower than *T_m,end_*, which is the usual behavior reported for a wide range of materials and measured with conventional DSC. The light blue area between *T_m,end_* and the transition temperature between *Domain II* and *Domain III* reflects the temperature range corresponding to *Domain IIb*, in which there are some crystal fragments (i.e., self-seeds) that are the responsible for the increment in nucleation density. However, for the sample that was kept for 300 s at *T_s_*, the transition to *Domain II* occurs at about 1 °C higher than *T_m,end_*; this is an unusual behavior that may result from the high heating and cooling rates employed.

Figure 4c illustrates how the width of *Domain II*, *Domain IIa* and *Domain IIb* vary as a function of the time spent at *T_s_*. The width of *Domain II* reduced with the time spent at *T_s_* due to the shift of the transition temperature between *Domain II* and *Domain III* to higher temperatures. In this case, the width of *Domain II* varied from 11 °C for 0.1 s holding time at *T_s_* to 7 °C for 300 s. This indicates that it is possible to fine tune the width of the *melt memory Domain* by varying the time spent at *T_s_*. This result implies that it would be possible to take advantage of this effect to reduce the time needed to process a polymer part.

### 3.3. Effectiveness of Self-Nuclei with Varying Cooling Rate

**Figure 5:**
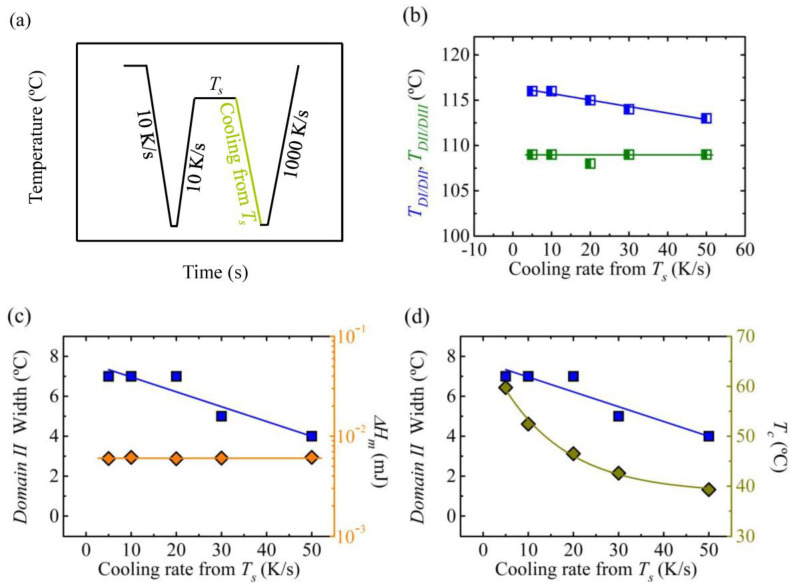
(**a**) Thermal procedure employed varying the cooling rate from *T_s_* temperature, in order to erase the thermal history the sample is kept at 160 °C for 30 s and it is cooled down to −50 °C, the sample is kept at this temperature, −50 °C, for 0.1 s, the sample is kept at *T_s_* temperature for 300 s, (**b**) transition temperature between *Domains* as a function of cooling rate from *T_s_*, (**c**) width of *Domain II* and melting enthalpy (when heating to *T_s_*) and (**d**) crystallization temperature cooling from *T_s_* as a function of cooling rate from *T_s_*.

When self-nuclei and/or self-seeds are produced in the sample, their effectiveness could change depending on the cooling rate employed to generate them. To investigate this effect, the thermal procedure depicted in Figure 5a was employed. In Figure 5b, the transition temperature between *Domains* is shown as a function of the cooling rate from the applied *T_s_* temperature. 

The transition temperature between *Domain II* and *Domain III* kept constant for the studied cooling rate range (5–50 K/s) according to Figure 5b. At higher cooling rates, the analysis of the results was complicated due to broadening of the crystallization peak. These results indicate that the annealed crystals (*DIII*) formed at *T_s_* were not sensitive to the cooling rates employed.

However, the transition temperature between *Domain II* (*self-nucleation Domain*) and *Domain I* (*melting Domain*) was reduced by about 3 °C (Figure 5b). To understand the mechanism lying behind this behavior, several parameters were considered. Figure 5c shows that the *Domain II* width was reduced from 7 to 4 °C when the cooling rate was increased from 10 to 50 K/s, nevertheless, the previous standard state (characterized with the melting enthalpy, which was directly correlated to the crystallinity level) was the same for all samples since only the cooling from *T_s_* was changed. Figure 5d shows how the crystallization temperature obtained during cooling from *T_s_*, decreased with cooling rate, as expected. 

The results presented in this section show that above a certain cooling rate there is a reduction in the width of *Domain II* or *self-nucleation Domain*. This means that at high cooling rates self-nuclei loose their effectiveness and lower *T_s_* temperatures are needed, i.e., a higher number of self-nuclei is required to induce an increase of the crystallization temperature. These results are in line with the ones reported by Jiang et al. [33]. They performed isothermal crystallization experiments with PBS and ideally self-nucleated PBS. This means that they employed the lowest temperature within *Domain II*, which can produce the maximum possible nucleation density without annealing (see ref. 3). Jiang et al. [33] showed that for self-nucleated PBS, there is a maximum crystallization rate at a certain undercooling, while for higher undercooling the crystallization rate advantage compared to the non-self-nucleated polymer is reduced, which indicates that at very high undercooling self-nuclei can lose their effectiveness. This issue was addressed recently by Fernández d’Arlas et al. [34] and Maiz et al. [35], proving that self-nuclei and nucleating agents, can lose their effectiveness when very high cooling rates are used. According to Fernández d´Arlas et al. [34] the self-nuclei are effective if the material, at the applied cooling rate, can undergo a significant crystallization process at high temperatures. The effect can be understood considering the higher nucleation density typically observed at lower crystallization temperatures, which demands for a higher number of self-nuclei (or nuclei induced by additives) to be present for their effect to be discernible.

As the crystallization peak broadened when the cooling rate was increased, which hindered an accurate analysis of the transition temperature between *Domain I* to *Domain II*, we decided to analyze the subsequent melting step after cooling the sample from the *T_s_* temperature. Even if it is not possible to investigate the different *Domains*, we could consider the melting enthalpy of the final heating scan to ascertain if the self-nuclei left in the sample are effective in crystallizing the sample or if when high cooling rates are used an amorphous sample is obtained, and how this depends on the employed *T_s_* temperature.

**Figure 6:**
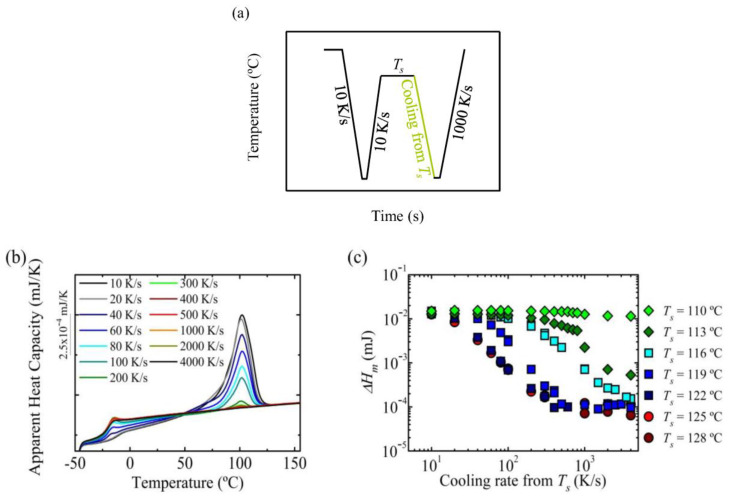
(**a**) Thermal procedure employed to analyze the effect of the cooling rate from *T_s_* temperature, in order to erase the thermal history the sample was kept at 160 °C for 30 s and it was cooled down to −50 °C, the sample was kept at this temperature, −50 °C, for 0.1 s, the sample was kept at the *T_s_* temperature for 300 s, (**b**) melting endotherm obtained at 1000 K/s after cooling the sample from a *T_s_* temperature equal to 119 °C at different cooling rates and (**c**) melting enthalpy as a function of cooling rate for samples kept at different self-nucleation temperatures.

Figure 6a illustrates the thermal procedure employed, which was identical to that employed in the previous experiments (Figure 5). The apparent heat capacity versus temperature curves (i.e., DSC heating scans) for the sample previously heated to *T_s_* = 119 °C are shown as a function of the cooling rate from the *T_s_* temperature, as an example, in Figure 6b. The melting peak reduced with increasing the cooling rate; for a cooling rate equal to 200 K/s, the peak was really small, and above 500 K/s, there was no crystallization in the previous cooling step.

Figure 6c shows the melting enthalpy, which is directly correlated to the crystallinity level, as a function of cooling rate from *T_s_* for different *T_s_* temperatures. For slow cooling rates, independent of the *T_s_* to which the sample was heated, all the data show similar melting enthalpy, which means that during cooling from *T_s_* the sample was able to develop similar crystallinity. Nevertheless, when the cooling rate increased, significant differences in the melting enthalpy could be observed depending on which *Domain* the sample was cooled from (considering the transition temperature between *Domains* previously reported for a cooling rate from *T_s_* equal to 10 K/s). 

When the sample was heated to *Domain I* or to high temperatures within *Domain II* at above 300 K/s, the sample was not able to crystallize, reaching melting enthalpy values below 1 × 10^−4^ mJ, which was the lowest value obtained for this sample at the highest possible cooling rate, thus it was considered that this value corresponded to an amorphous sample. The presence of some self-nuclei, obtained by heating the sample to the high temperature region within *Domain II*, were not able to increase the melting enthalpy, and similar values to the sample heated to *Domain I* were obtained in Figure 6c. However, when the sample was heated to the lowest temperatures within *Domain II*, the presence of self-nuclei enhances the melting enthalpy when cooling rate was increased. For example, in the case of the sample heated to 116 °C, which is the lowest temperature within *Domain II*, an enthalpy of 3 × 10^−3^ mJ was obtained at 500 K/s cooling rate, whereas the sample heated to higher *T_s_* temperatures were completely amorphous or have a negligible enthalpy.

When the sample was heated to *T_s_* temperatures that correspond to *Domain III*, in which the crystal fragments left (i.e., self-seeds) were able to anneal, it can be observed that for temperatures close to *Domain II* (e.g., *T_s_* = 113 °C)*,* the amount of those crystals was really low, since the enthalpy was 5 × 10^−4^ mJ. However, when lower *T_s_* temperatures (e.g., *T_s_* = 110 °C) were employed, the amount of molten crystals was small, observing only a slight reduction in the melting enthalpy at the highest cooling rate, which was practically negligible.

Overall, the experiments presented in this section show that the effectiveness of self-nuclei depends on the applied cooling rate. When only some self-nuclei were left, at temperatures within *Domain II* but close to *Domain I*, the results were the same as the sample cooled down from *Domain I*. When a higher number of self-nuclei and probably self-seeds were left, at low temperatures within *Domain II*, the melting enthalpy increased in comparison with the sample cooled from a homogenous melt state. From these results, it could be concluded that to have a significant increase in melting enthalpy, a high density of self-nuclei and self-seeds was required.

### 3.4. Effect of the Previous Standard State on the Self-Nucleation Domains: Varying the Cooling and Heating Rates 

Considering the kinetic nature of self-nucleation, the temperature range corresponding to different *Domains* should depend on the previously formed semicrystalline standard state. Although in literature, this has been mentioned in several works [2,3,4], there are not very detailed studies focusing on how the semicrystalline standard state can affect self nucleation. Alamo et al. [14,36] have considered the effect of the crystallinity degree on melt memory for random ethylene 1-butene copolymers of different molecular weights. In order to create samples with different degrees of crystallinity, the samples were heated to different temperatures [14] or they were isothermally crystallized at different temperatures [36].

**Figure 7:**
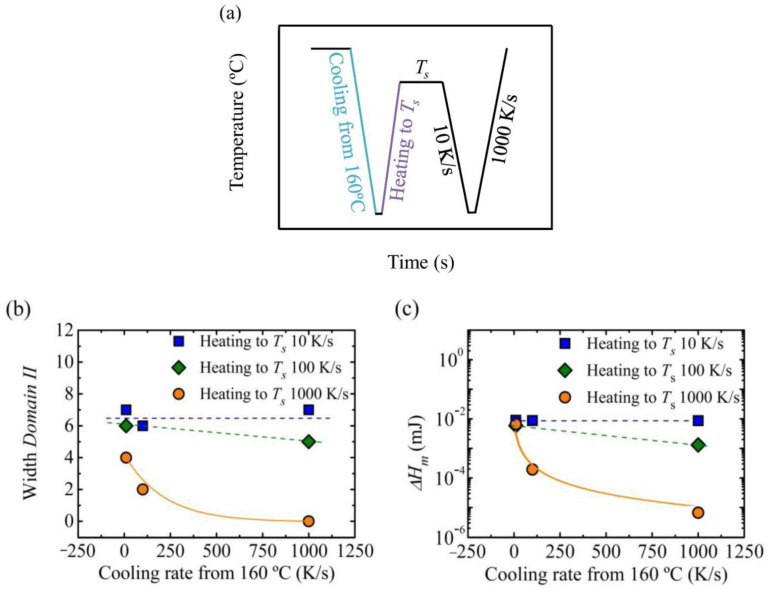
(**a**) Thermal procedure employed to analyze the effect of cooling rate from 160 °C and heating to *T_s_*, in order to erase the thermal history the sample is kept at 160 °C for 30 s and it is cooled down to −50 °C, the sample is kept at this temperature, −50 °C, for 0.1 s, the sample was kept at the *T_s_* temperature for 300 s, (**b**) width of *Domain II* as a function of the cooling rate from 160 °C and (**c**) melting enthalpy as a function of the cooling rate from 160 °C.

In this work, we analyzed the effect of the cooling rate from the isotropic melt (i.e., during this cooling, the standard semicrystalline state was formed) and the subsequent heating rate to *T_s_* on the *self-nucleation Domains*, as a different method for the variation of the standard crystalline state. We employed three different rates: 10, 100 and 1000 K/s. The thermal protocol employed is depicted in Figure 7a.

Figure 7b shows the width of *Domain II* as a function of cooling rate. The samples were cooled from a *T_s_* = 160 °C (from the isotropic melt or *Domain I*). It can be observed, that when the heating rate to *T_s_* was maintained constant at 10 K/s, the width of *Domain II* did not change. From these results, we could conclude that the width of *Domain II* was related to the melting enthalpy of the sample, and thus to the crystallinity level, when it was heated to the *T_s_* temperature, which was also constant (see Figure 7c). When the sample was cooled down at 1000 K/s (Figure 7b), during the subsequent heating at 10 K/s to the *T_s_* temperature, cold crystallization occurred, so when the sample reached the *T_s_*, the melting enthalpy was the same. However, when the sample was heated to the *T_s_* temperature at 1000 K/s a different behavior was observed, as a reduction of the width of *Domain II* was observed from 4 to 0 °C. For a heating rate of 100 K/s to *T_s_*, an intermediate behavior was obtained in Figure 7b, with a slight reduction of the width of *Domain II*. 

The reduction of *Domain II* width in Figure 7b is related to the decrease in the melting enthalpy, as can be seen in Figure 7c, as the same trend is observed for both quantities. When high heating rates were employed to heat the sample to the *T_s_* temperature, the chains did not have enough time to reorganize and there was no cold crystallization, so a lower crystallinity degree (i.e., lower melting enthalpy) was obtained in comparison with the reference or standard conditions that corresponded to 10 K/s cooling/heating rate. If a vertical line is drawn in Figure 7b, the effect of the heating rate to *T_s_* could be observed keeping constant the cooling rate from 160 °C. In all cases, the *Domain II* width decreased when the heating rate increases, but the decrease was much higher at higher cooling rates.

Although the melting enthalpy was only considered, probably employing high cooling and heating rates the thickness of the crystals could also be reduced, and this should result in narrower *Domain II* since the melt memory effect could be erased without increasing too much the superheating, as the crystals are more metastable (i.e., thinner lamellae).

Chen et al. [14] have studied the effect of crystallinity degree on random ethylene 1-butene copolymers with different molecular weights that contain 2.2% of branches. The authors observed that for copolymers with a low molecular weight, 16,000 g/mol, a crystallinity degree of 24.8% is required in order for the copolymers to show melt memory effects. However, increasing the molecular weight, the crystallinity degree required to obtain melt memory effects is reduced until 0.6% for the 420,000 g/mol sample. For random copolymers the origin of melt memory results from the complex topology formed in the melt, this topology hinders the diffusion of chain segments to obtain an isotropic or homogeneous melt. For samples with high molecular weight, a small amount of crystals was enough to result in melt memory since a high entanglement density was obtained, which hindered the “dissolution” of self-nuclei. On the contrary, for samples with low molecular weight, there is a low entanglement density, which facilitated the diffusion of chains and therefore the “dissolution” of self-nuclei. 

In this work, a linear homopolymer was analyzed and, thus, the melt memory effect did not come from complex chain topologies but from the presence of intersegmental interactions between the chain segments that were forming the crystal. If the sample contained a higher amount of crystals, the number of interactions between the chain segments previously in the crystals was higher and thus resulted in larger melt memory effects.

**Figure 8:**
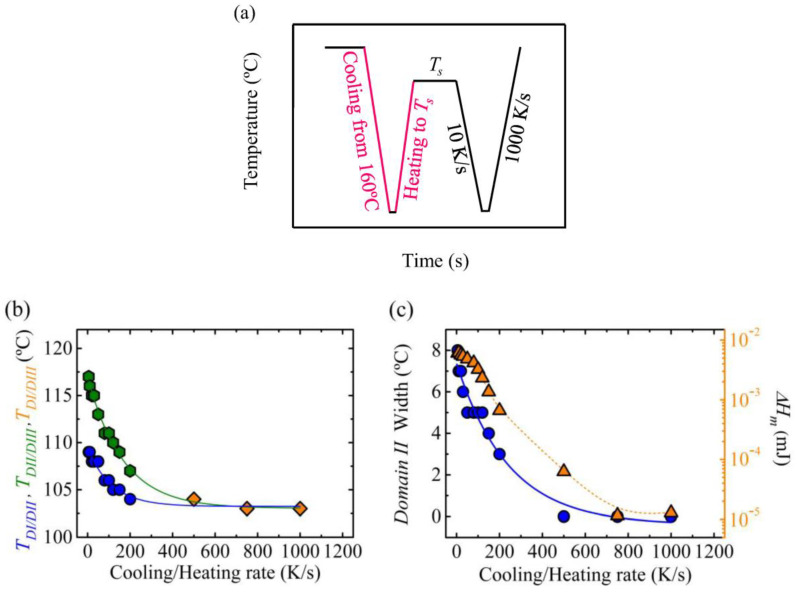
(**a**) Thermal procedure employed to analyze the effect of cooling rate from 160 °C and heating to *T_s_* (the same rate is employed for cooling and heating in each set of experiments), in order to erasure the thermal history the sample is kept at 160 °C for 30 s and it is cooled down to −50 °C, the sample is kept at this temperature, −50°C, for 0.1 s), the sample is kept at the *T_s_* temperature for 300 s, (**b**) transition temperature between *Domains* as a function of the cooling/heating rate and (**c**) width of *Domain II* and melting enthalpy as a function of the cooling/heating rate.

Considering that the key parameter that determines the width of *Domain II* is the melting enthalpy (i.e., crystallinity level) obtained during the subsequent heating to the *T_s_* temperature (after having created the standard semicrystalline state by cooling from the isotropic melt), it was decided to employ the same rates to cool down the sample from 160 °C and to subsequently heat it to the *T_s_* temperature for self-nucleation, with the aim of covering a wide range of melting enthalpies. The thermal procedure employed is shown in Figure 8a. This procedure allowed us to obtain materials with enthalpies that cover 3 orders of magnitude, from 1 × 10^−2^ to 1 × 10^−5^ mJ. 

The transition temperature between *Domains* was reduced as the cooling/heating rate was increased, see Figure 8b. For rates equal or above 500 K/s there was no *Domain II*, and the sample went directly from *Domain I* to *Domain III*. In Figure 8c, the width of *Domain II* and the melting enthalpy, proportional to the crystallinity level, as a function of the heating/cooling rate are depicted. A drastic reduction of both parameters with the increase of cooling/heating rate can be observed. Our results demonstrated the importance of the melting enthalpy (directly proportional to the crystallinity degree) of the sample when it reached the *T_s_* temperature on the width of the *self-nucleation Domains*. 

### 3.5. Effect of the Previous Standard State on the Self-Nucleation Domains: Thickening of Crystals by Successive Self-Nucleation and Annealing (SSA)

With the aim of creating thicker crystals while increasing the crystallinity degree, the successive self-nucleation and annealing (SSA) technique, designed by Müller et al. [37,38] and recently reviewed [39], was applied. The idea is to thermally fractionate the sample by SSA before melting the crystals produced, to see if the thickness and stability of the previously existing crystals influence the memory effects exhibited by the sample.

SSA is a thermal fractionation technique based on the application of a special thermal protocol that promotes molecular segregation during crystallization and annealing designed to fractionate the material by crystallizable sequence lengths. It is particularly sensitive to defects like branches along the chains, comonomer units, stereo-defects, etc. Once SSA is applied to a polymer sample, the final heating of the material shows several melting peaks, each one corresponding to a different thermal fraction. This distribution of melting points also reflects a distribution of lamellar thickness in the sample provoked by SSA thermal fractionation. This technique has been very useful to characterize ethylene/alfa-olefin copolymers, polypropylenes, block copolymers and more recently, segmented thermoplastic polyurethanes, recycled polyolefin blends, copolyesters and polysulfide based copolymers [39,40,41,42,43,44]. 

**Figure 9:**
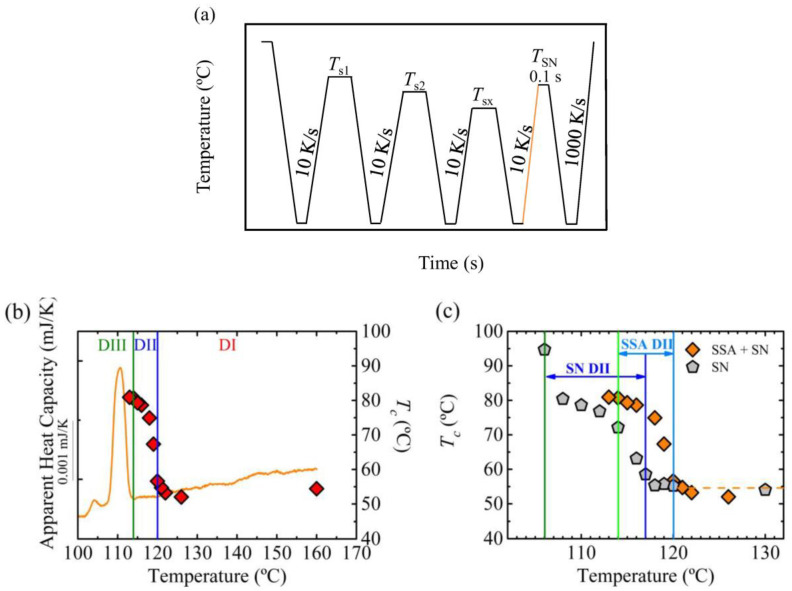
(**a**) SSA thermal procedure applied followed by SN, in order to erase the thermal history, the sample is kept at 160 °C for 30 s and then it is cooled down to −50 °C, the sample is kept at this temperature, −50°C, for 0.1 s, the sample is kept at the *T_s_* temperature for 0.1 s; (**b**) DSC heating scan (in orange color) of the sample submitted to SSA followed by SN and the corresponding crystallization temperature as a function of *T_s_* temperature and (**c**) crystallization temperature of the sample submitted to standard SN and the sample submitted to SSA followed by the SN procedure as a function of the self-nucleation temperature. Vertical lines correspond to the transition temperatures between *Domains*.

Thermal fractionation in homopolymers is based on the molecular fractionation due to differences in chain length and depends on their molecular weight dispersity and melt viscosity. The fractionation produced by SSA in linear crystallizable homopolymers without defects in their chains is less effective than with copolymers (or any polymeric material with intrachain defects). However, it can be used also to efficiently anneal polymeric crystals, since SSA technique consists of applying successive self-nucleation and annealing steps. The *T_s_* temperature was reduced by 5 °C in each step, so when *T_s_* was reduced, the crystals that were not able to melt underwent annealing. The success of the applied SSA procedure to PBS sample can be observed by analyzing the final heating scan (in orange in Figure 9a), which shows the melting of the produced fractions, which corresponds to the melting of crystals of different lamellar thickness. The melting temperature of the highest melting fraction was higher than that of the sample summited to a standard non-isothermal crystallization procedure, as could be expected, which reflects that the procedure has been effective to anneal and thicken the crystals. We have previously shown that SSA could be effectively applied by fast scanning calorimetry [45]. 

After applying SSA, the sample was heated to the selected *T_s_* temperature and kept at this temperature for 0.1 s. Each time that a new *T_s_* was analyzed, first the SSA protocol was applied to the sample: the thermal procedure employed is depicted in Figure 9a. The crystallization temperature corresponding to each *T_s_* temperature on top of the melting endotherm is shown in Figure 9b, in this plot the transition temperatures of the *Domains* are shown as well. It is interesting to compare the results obtained applying the standard SN procedure (keeping the sample 0.1 s at *T_s_* temperature in both cases) and the SN procedure after SSA. Figure 9c shows that when SSA was applied, self-nuclei survive until higher temperatures, 120 °C, in comparison with 117 °C for standard SN. The results can be explained by considering that SSA induced the formation of thicker crystals, which therefore require higher temperatures to transform to the isotropic melt state. Annealing of the crystals, i.e., *Domain III*, was observed as well at higher temperatures for samples submitted to SSA before SN, 114 °C, whereas when standard SN was applied *Domain III* appeared at 106 °C. As expected, thicker crystals (which melt at higher temperatures) could anneal at higher temperatures. 

From the final melting endotherms, the melting enthalpies obtained by both protocols were measured. When a standard SN procedure was applied during the heating to *T*_s_ temperature, the enthalpy was equal to 7.67 × 10^−3^ mJ, whereas when SSA was applied and then the sample was heated to *T_s_*, the melting enthalpy was equal to 1.65 × 10^−2^ mJ, this means that SSA was very effective in increasing the crystallinity degree. 

From the melting temperature it is possible to estimate the lamellar thickness employing the Gibbs-Thomson equation,

(1) $$T_{m}\;=\;T_{m}{}^{0}\;\left(1-\frac{2\sigma_{e}}{∆h_{f}^{0}l}\right),$$

where *T_m_*^0^ is the equilibrium melting point, *σ_e_* is the chain folded surface free energy and $∆h_{f}^{0}$ is the bulk enthalpy of melting per unit volume. For this equation the equilibrium melting point can be obtained considering a lamella with an infinite thickness, i.e., 1/l equal to 0 [46,47]. Considering the data obtained by Arandia et al. [40] for the same PBS used in this work, the lamellar thickness of the PBS has been estimated when a standard SN procedure and SSA followed by SN procedure have been applied. For standard SN a melting point of 94 °C was obtained, which corresponded to an approximate lamellar thickness of 3.4 nm, whereas when SSA was applied a significant increase in lamellar thickness occurred, obtaining a lamellar thickness of 4.9 nm (melting point 111 °C). These results supported that the shift of the *Domains* to higher temperatures for the sample that had undergone SSA procedure before SN corresponded to an increment of lamellar thickness and also to an increase in the degree of crystallinity.

## 4. Conclusions

In this work, the parameters affecting the melt memory effect were studied employing PBS and taking advantage of the high cooling/heating rates and short times accessible with flash DSC. For the first time it was proved that although short times spent at self-nucleation temperature did not affect the crystallization temperature (nucleation density), time spent at *T_s_* did affect the width of *Domain II*. The effect of the cooling rate from *T_s_* temperature on different *Domains* was investigated, observing a reduction of *Domain II* with increasing the rate. In addition, these experiments revealed that a high density of self-nuclei and self-seeds are needed to induce the crystallization of the material when high cooling rates are applied. The effect of the previous standard state on melt memory was analyzed, providing a correlation of the width of *Domain II* and melting enthalpy/crystallinity level of the sample at *T_s_*. Moreover, the SSA technique was applied to thicken the crystals and increase the crystallinity degree, which resulted in a shift of transition temperature between *Domains* to higher temperatures in comparison with the standard state sample.

Overall, this work shed lights on the kinetic nature of *self-nucleation Domains* and provided an example to fine tune the thermal properties of polymers, which can be extended to other semicrystalline material. Considering the short times and high cooling/heating rates involved in industrial processing of materials, this work can help to reduce the time needed to produce plastic parts and modify the thermal properties of polymer ad hoc for each application.
